# Nonpuerperal Uterine Inversion: What the Gynaecologists Need to Know?

**DOI:** 10.1155/2020/8625186

**Published:** 2020-06-01

**Authors:** R. P. Herath, M. Patabendige, M. Rashid, P. S. Wijesinghe

**Affiliations:** ^1^Department of Obstetrics and Gynaecology, Faculty of Medicine, University of Kelaniya, Kelaniya, Sri Lanka; ^2^University Obstetrics and Gynaecology Unit, Teaching Hospital, Mahamodara, Galle, Sri Lanka; ^3^Sidra Medicine, Doha, Qatar

## Abstract

**Introduction:**

Nonpuerperal uterine inversion (NPUI) is a rare clinical problem with diagnostic and surgical challenges. The objective of our study was to review the literature on NPUI and describe causative pathologies, diagnosis, and different surgical options available for treatment.

**Materials and Methods:**

A comprehensive literature review was carried out on MEDLINE and Google Scholar databases to look for NPUI using the term “non-puerperal uterine inversion,” and further went through the cross-references of the published articles. Data are published case reports from 1911 to September 2018. Of the 153 published cases, 133 reports had adequate details of surgery for analysis. These reports were analyzed, concerning the clinical presentation, methods of diagnosis, and surgical treatment.

**Results:**

Mean age of the women was 46.3 years (standard deviation: 18, *N* = 153). Leiomyoma remained the commonest (56.2%) aetiology. While malignancies contributed to 32.02% of cases, 9.2% were idiopathic. High degree of clinical suspicion and identification of unique features on ultrasonography and magnetic resonance imaging enable prompt diagnosis. In cases of uncertainty, laparoscopy or biopsy of the mass was used to confirm the diagnosis. Hysterectomy or repositioning and repair of the uterus are the only treatment options available. The surgical methods implemented were analyzed in three aspects: route of surgical access, method of repositioning, and final surgical procedure undertaken. The majority (48.8%) had only abdominal access, while 27.1% had both abdominal and vaginal access. Haultain procedure was the most useful procedure for reposition (18.0%) of the uterus. The majority (39.7%) required abdominal hysterectomy with or without debulking of the tumour abdominally, while 15.0% had uterine repair after repositioning. We reviewed the different surgical techniques and described and proposed a treatment algorithm.

**Conclusions:**

Fibroids were the commonest cause for NPUI. Malignancies accounted for one-third of cases. A combined abdominal and vaginal approach, followed by hysterectomy or repair after repositioning, seems to be better for nonmalignant cases.

## 1. Introduction

Uterine inversion is a condition where the fundus of the uterus turns inside out and the latter prolapses through the cervix. Puerperal uterine inversion was the first uterine inversion type to be recognized, possibly due to its common occurrence. In Ayurveda, the ancient Hindu system of medicine, there is some evidence to suggest that uterine inversion was known to them. However, Hippocrates (460–370 B.C.) is credited as the first to recognize uterine inversion [1, 2].

Inversion of the uterus was classified by Jones in 1951 into two types: puerperal or obstetric and nonpuerperal or gynaecological [3]. While puerperal inversions are seen following delivery or miscarriages and may be acute or chronic, the nonpuerperal variety is mostly related to benign or malignant tumours associated with the uterine corpus. Nonpuerperal inversions present mostly as chronic cases, although Das has reported 8.6% of nonpuerperal inversion as a sudden onset [1].

Nonpuerperal uterine inversion (NPUI) is rare, and actual incidence is not known. Most of the published literature on NPUI is in the form of case reports. We analyzed the possible causes and the treatment options used by the attending gynecologists in NPUI. To our knowledge, this is the first review to analyze the success rates of the treatment options used in cases of NPUI.

Even though the mechanism of obstetric uterine inversion is well understood, the mechanism of nonpuerperal inversion is not so clear. If the myometrium becomes distended due to a tumour within the cavity, it becomes irritable and initiates expulsive contractions, which can dilate the cervix and assists in the expulsion of the tumour, dragging its fundal attachment. The weight of the tumour, manual traction on the tumour, or increased intra-abdominal pressure due to coughing, straining, and sneezing may also contribute to NPUI [1, 2]. With sarcomas, the area of the uterine wall weakened by the growth is believed to prolapse into the cavity and thus be brought under the influence of the active uterine musculature.

## 2. Materials and Methods

A comprehensive literature review found that, in 1911 and in 1940, Thorn et al. and Das et al. have reported 96 and 54 cases of NUPI, respectively [1, 4]. We reviewed the literature from inception, published since the work of Thron et al. in 1911, till September 2018 in MEDLINE and Google Scholar databases using the term “non-puerperal uterine inversion” and further went through the cross-references of the published articles.

## 3. Results

Our literature search found 153 cases published since the publication of Das, accounting for 303 cases in total (Table 1). The mean age of the women was 46.3 years (SD-18) with a range of 14–88 years.

### 3.1. Causes of NUPI

Leiomyoma was the commonest cause for NPUI found in our study accounting for 86 (56.2%) of the case [5–86]. The nomenclature used by different authors to describe the causative pathology showed a big variation as the reported cases are published over many decades. We tried to group them into the following categories depending on available information. Carcinomas [87–99] and sarcomas [3, 24, 100–109] accounted for 13 (8.5%) each, while mixed Mullerian [106, 110–118] contributed to 10 (6.5%) cases.

Rare causes such as fibrosarcoma [119], epidermoid carcinoma [120], endometrial sarcoma [121, 122], carcinosarcoma [123], rhabdomyosarcoma [124–127], endometrial polyp [127, 128], immature teratoma [129, 130], combination of fibroid and a cervical carcinoma [131], and pelvic organ prolapse [132, 133] contributed to 17 (11.1%) cases of our study. There was no obvious cause for further 14 (9.2%) cases [134–147] (Table 1). Turan et al. reported a case of cervical inversion without uterine inversion, which was not included in the study [148]. We noted that 49 out of 153 (32.02%) cases were due to malignancies.

## 4. Discussion

### 4.1. Clinical Presentation and Diagnosis

Most women presented with foul-smelling vaginal discharge or irregular vaginal bleeding, some to the extent of causing anaemia needing blood transfusions [78]. There might be abdominal cramps, pelvic discomfort, and fullness of the vagina or pressure in the vagina. Chen et al. reported a case presenting as worsening dysmenorrhoea, menorrhagia, and dyspareunia. Acute urinary retention, needing suprapubic catheterization, has been described following NPUI [20]. Hypovolaemic shock has been reported in a case of acute uterine inversion due to a fibroid [16].

**Figure 1:**
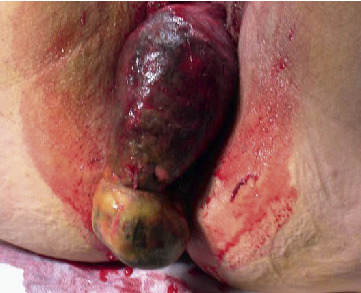
Macroscopic appearance of a complete nonpuerperal uterine inversion due to a fibroid [34].

The inverted uterus forms an inverted pyriform swelling, which occupies the upper part of the vagina. In the case of total inversion, the mass will be protruding out of the introitus. It is smooth, dark red, and usually bleeds on palpation (Figure 1) [34]. If the tubal ostia is seen, it is conclusive of uterine inversion, but if the mass is infected or sloughing, ostia may not be easily seen [144].

Lascarides in 1968 described three important clinical signs in the diagnosis of NPUI: first, the cervical ring may not be recognizable along the proximal part of the mass; second, one cannot find the opening of uterine cervix or probe the endometrial cavity; third, rectal examination reveals that the uterus is not in its normal position in the pelvis, and the cupping of the fundus can sometimes be palpable [72]. It needs to be highlighted that the cervical ring is not identified only in cases of complete and total inversions. Furthermore, there are reported cases where the diagnosis of chronic NPUI was overlooked, and excision of the “vaginal mass” resulted in severing the fundus from the uterus and inadvertent entering into the peritoneal cavity [149].

NPUI has been classified into three groups depending on the degree of the inversion [12] (Table 2).

### 4.2. Imaging

#### 4.2.1. Ultrasonography

Ultrasonography (USS) should be the first line of investigation considering availability and simplicity. USS can help with both the diagnosis of NPUI and diagnosis of its aetiology. Sonographic characteristics of “Y”-shaped uterine cavity, in the longitudinal plane are seen in incomplete uterine inversions. The base of “Y” is the noninverted endometrial lining. In contrast to incomplete inversion, the longitudinal view in complete inversion shows a “U”-shaped configuration, with the limbs of the “U” representing the complete inverted endometrial lining extending both anteriorly and posteriorly [42]. Some authors described the “target” sign while imaging the lower pelvis in the transverse plane, with the hyperechoic fundus surrounded by a rim, representing fluid within the space between the inverted fundus and the vaginal wall [11, 108].

During an ultrasound examination, leiomyomas usually appear as well-defined, solid, concentric, hypoechoic masses that cause a variable amount of acoustic shadowing [150]. However, the ultrasound observation of a large mass, with inhomogeneous structure, without acoustic shadowing, and with rich central vascularization, assessed with colour Doppler has proven to be suspicious of malignancy [151]. On ultrasound, sarcomas typically appear as isolated large solid masses with inhomogeneous echogenicity of the solid tissue, sometimes containing cystic (usually irregular) areas and usually not having shadowing or calcifications [152, 153].

The 3D power Doppler with USS has been used more recently in the diagnosis of NPUI as it can clearly show the changes in the uterine artery course in relation to the uterine body. Zohov et al. emphasized that once the vaginal probe (with 3D power Doppler) is applied directly to the uterine corpus of inverted uterus, it showed bilateral uterine arteries in a longitudinal central location along the uterine body, with a U-turn sign, showing a central course of the main uterine vessels instead of their normal anatomical peripheral location laterally alongside the corpus of the uterus [81]. Ultrasonography has its limitations, the main being its operator dependency. In cases of NPUI, many reports of overlooking the diagnosis have been reported in the literature [33, 53, 80]. Despite above, Atalay et al. have emphasized the limited diagnostic value of transvaginal ultrasonography, in cases with large masses protruding into the vagina [33].

#### 4.2.2. Magnetic Resonance Imaging (MRI)

**Figure 2:**
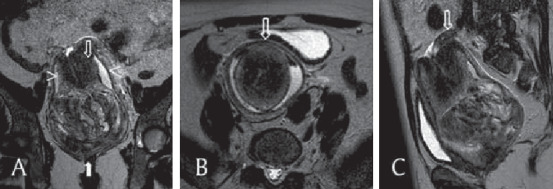
T2-weighted MRI of complete nonpuerperal uterine inversion due to fibroid. (a) This coronal image shows a vaginal heterogeneous mass (filled arrow), with the uterine corpus in a U-shape above the mass (empty arrow). The cervix surrounds the corpus, and the vaginal fornix surrounds both the corpus and the cervix (arrowheads). (b) This axial image shows, from the center outwards, the uterine corpus, the cervix, and the fornix and the invaginated round ligaments in a bullseye appearance (arrow). (c) This sagittal image shows one ovary above the cervix (arrow) [74].

MRI is found to be sensitive in the diagnosis of NPUI. The distinct observations identified are U-shaped uterine cavity, a thickened and inverted uterine fundus on a sagittal section, and a “bull's eye” configuration on the horizontal section [93, 142]. U-shaped cavity could also be noted even in cases of inversion due to pedunculated tumours [93]. In complete inversion, identifying the round ligaments and fallopian tubes protruding centrally from the top of the uterus will help in arriving at the diagnosis (Figure 2) [74, 93, 95, 142]. In case of a malignancy causing NPUI, MRI will further help in imaging lymph nodes.

#### 4.2.3. CT Scan

CT scan has not been very useful in the diagnosis of NPUI. Especially in postmenopausal patients, inversion can be misdiagnosed as a cervical malignancy. But nonvisualization of the uterus and visualization of a low-density material in the middle of the pelvis due to oedematous endometrium and myometrium are features suggestive of uterine inversion. It can be an option in situations where MRI is not possible. The contrast-enhanced examination is favored for delineation [37].

### 4.3. Examination under Anaesthesia, Laparoscopy, Frozen Section, and Biopsy

In most of the case reports we reviewed, the difficulties of clinical diagnosis and interpretation of ultrasonography have been emphasized [53]. To overcome this, examining under anaesthesia and histological sampling of the vaginal mass have been suggested [53]. Demonstrating the endometrium on the surface of the mass will be confirmatory of the diagnosis. In our analysis, we found that 32.02% of NPUI were associated with malignancies. Therefore, histological evaluation of the mass is justifiable, before the definitive surgery, unless the causative pathology of a fibroid is obvious.

**Figure 3:**
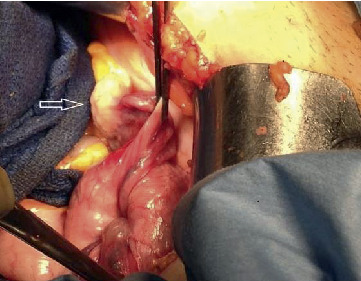
The appearance of ovaries and tubes projecting out of the indented uterine fundus has been described as the “flower vase appearance” in cases of nonpuerperal uterine inversion [74].

Viewing the pelvis at laparoscopy or laparotomy is an alternative way to confirm NPUI if the imaging modalities fail to provide a reasonable diagnosis. The appearance of ovaries and tubes projecting out of the indented uterine fundus has been described as the “flower vase appearance” in cases of NPUI (Figure 3) [74].

### 4.4. Treatment of NPUI

**Figure 4:**
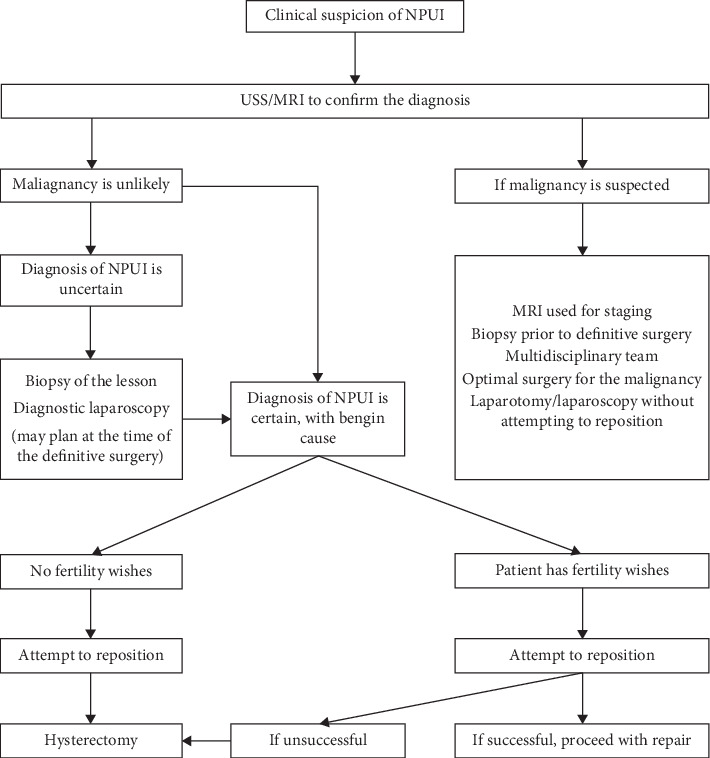
Treatment algorithm of nonpuerperal uterine inversion.

Initial assessment and resuscitation would be the priority as some patients may be in septic or in haemorrhagic shock, followed by correction of anaemia, pain relief, and starting antibiotics. Once stabilized, all steps should be followed to confirm the diagnosis and to establish the possible aetiology. The type and approach of surgery should be individualized considering the age, desire for future fertility, aetiology, and the stage of the disease in case of malignancy. We propose the guide given in the algorithm in Figure 4 to investigate and plan treatment.

If any uncertainty of the diagnosis exists after imaging, it should be cleared with laparoscopy and biopsy before definitive surgery is performed. If the biopsy confirms a malignancy, multidisciplinary involvement is important to plan the optimum treatment for the given type and stage of the malignancy.

Surgery is the mainstay of treatment of NPUI, focusing on the repositioning of the uterus. Repositioning is essential if uterine preservation is considered, as it is the only way to prevent pain, bleeding, infections, and gangrene. It should be assumed that hysterectomy would be technically easier on a normally positioned uterus, rather than the inverted uterus, as repositioning would restore normal anatomy with which gynecologists are familiar with.

While stage 1 inversion will often offer easy repositioning of the fundus, inversions of stages 2, 3, and 4 are likely to be more demanding. Exclusion of malignancy and excision of the causative benign tumour are essential before repositioning and repair. If repositioning is impossible, the only option left would be hysterectomy. Authors believe that attempting to reposition a uterus with a malignancy would be detrimental as the peritoneal cavity would be exposed to the pathology through the incised uterine wall.

Over many decades, authors have attempted different surgical options to solve NPUI, probably the first time they attended such a case. Only 133 out of 153 cases had details regarding the surgical management, and each of these 153 cases was analyzed for the suitability after careful reading by the authors. After analyzing these 133 cases where surgical details were available, we recognized three main aspects to consider: (1) route of surgical access (either abdominal, vaginal, or both), (2) attempt to reposition, and (3) planning of the eventual surgical procedure (resection of the causative tumour and repair or hysterectomy) as shown in Table 3.

When there is a mass protruding out of the vagina, it is tempting for the gynecologist to consider vaginal approach. The vaginal approach was selected by 24 (18.0%) surgeons, while 65 (48.8%) surgeons have performed a laparotomy. The combined abdominal and vaginal approach was preferred by 36 (27.1%) (Table 3). Most of the surgeons who opted for the latter have attempted the vaginal route first and subsequently went on to perform a laparotomy to complete the procedure. More recently, the laparoscopic approach has been used for diagnosis and as safety tools while performing vaginal hysterectomy [52]. The authors noted that most of the recent reports preferred either abdominal or combined approaches.

Techniques of repositioning uterus such as Huntington, Haultain, Spinelli, and Kustner's operations were initially described for treating puerperal inversions. Subsequently, surgeons have used the above procedures to treat NPUI also. Haultain procedure seems to be the most successful method to achieve repositioning (18.0%, 24 out of 133 cases) (Table 3). In 52.3% cases, either the repositioning attempt failed or there was no mention of an attempt to reposition. Huntington procedure does not seem to be very successful in NPUI. Subtle variations of surgical techniques have been applied by surgeons to reposition the uterus. Table 3 summarizes the surgical methods used by previous authors to reposition the uterus and outcomes of the surgery.

A large proportion (39.8%) of women underwent abdominal hysterectomy, while further 15.8% underwent vaginal debulking of the tumour followed by abdominal hysterectomy. An additional 19.6% underwent a vaginal hysterectomy. After repositioning, 15.0% underwent uterine repair.

### 4.5. Abdominal Approaches

Huntington procedure involves laparotomy, locating the cup of uterus formed by the inversion, dilating the cervical ring digitally, and gentle upward traction of the round ligaments and the fundus of the uterus, to reposition the uterus. Nicol Haultain in 1908 described a procedure to replace puerperal uterine inversions. Following laparotomy, the inversion ring is incised posteriorly to facilitate repositioning with traction on the fundus [154]. Lai et al., in their case, repositioned the uterus during laparotomy by making an incision on the anterior aspect of the cervical constriction ring in contrast to Haultain procedure where the incision is made on the posterior ring [9].

Tjalma et al. described abdominal retroperitoneal dissection of ureters and uterine arteries, before progressing to hysterectomy after opening the vaginal wall anteriorly. They emphasized that identification of the ureter, in these cases where distorted anatomy is the hallmark, will minimize ureteric injuries [112]. Sharma et al. described abdominal resection of fibroid and abdominal hysterectomy. Round ligaments were clamped and cut followed by cornual fundal structures. After ligating the uterine arteries, the uterus was cut open with a midline vertical incision to remove the fibroid removed, and hysterectomy was completed as usual [26].

Skinner et al. reported a case of NPUI due to 6 cm atypical leiomyoma in a 27-year-old woman who was committed to retaining fertility. The condition was confirmed at laparoscopy, and repositioning was attempted but failed. She was given three doses of gonadotrophin releasing hormone (GnRH) analogue, over three months to shrink the fibroid. Three months later, the leiomyoma, which was 3 cm by now, was resected vaginally using an electrosurgical loop. Yet the uterus could not be repositioned and ended up in abdominal hysterectomy. Authors suggest that the pretreatment with the GnRH analogue made it difficult to reposition the uterus, thus increasing the resistance to dilate the cervix, although it made the fibroid smaller [12]. De Vries reported that they noted an accidental entry into the peritoneal cavity while trying to debulk the vaginal mass. Subsequently, they performed a laparotomy for safety and completion of the repair of the defect [29].

Krissi et al. performed a laparoscopy to confirm the diagnosis of NPUI before proceeding to vaginal myomectomy and subsequent laparotomy. He incised the constricting ring both anteriorly and posteriorly before repositioning the uterus [36]. Laparoscopic repositioning and repair were reported by Zhang et al. after releasing the anterior cervical ring during laparoscopy. They performed a vaginal myomectomy before repositioning [69].

### 4.6. Vaginal Approaches

In Kustner'ss operation, the pouch of Douglas is opened by posterior colpotomy, and the posterior uterine wall is incised. The surgeon's thumbs make pressure upon the rear wall of the uterus leading to reversion and restoring it to its normal position within the pelvis. The corpus is flipped through the posterior colpotomy, and the incision in the posterior uterine wall is repaired, having trimmed any myometrium if necessary, to achieve reapproximation of the serosal surface. The uterus is replaced within the pelvis, and the colpotomy is closed [15]. Alumi et al. noted that Kustner's procedure alone did not give enough space to reposition the uterus, and had to extend the incision along the posterior vaginal wall [146].

The Spinelli operation is similar in principle to the Kustner's operation, except that the incision into the uterine wall is made anteriorly after the bladder has been retracted upwards [14]. The uterus is then repositioned as in Kustner's operation. Once the repositioning has been done, uterine incision can be repaired or vaginal hysterectomy can be accomplished with the uterus in its anatomical position. Fofie and Baffoe reported a case with a slight modification to the Spinelli operation, where they had to extend the incision along the anterior uterine wall over the fundus to help reposition [30].

Mwinyoglee et al. reported an NPUI which was treated with vaginal hysterectomy without repositioning the uterus [106]. They used ultrasonography to locate the bladder in the cervico-forniceal region before making the incision. Once the bladder was safely dissected and pushed up, the uterosacral ligaments, cardinal ligaments, and the uterine arteries were dealt with in the standard manner. Then, they bisected the corpus to access the upper pedicles and completed the hysterectomy. They left the vaginal wall unclosed considering the gross edema.

Mayadeo and Tank , in 2003, described a case of incomplete lateral inversion of the uterus, diagnosed at laparoscopy, and treated with vaginal hysterectomy without repositioning the fundus [13]. In this case, as the inversion is incomplete, the lower pedicles could have been reached as in a routine vaginal hysterectomy. Herath et al. reported a case where they performed a vaginal hysterectomy under direct observation with a laparotomy [34]. Simms-Stewart et al. who treated a postmenopausal NPUI with subtotal vaginal hysterectomy did not attempt to remove the cervix considering the risks associated with the distorted anatomy [97]. The number of surgical modifications used in treating NPUI highlights the vast diversity of clinical presentations and surgical difficulties encountered by the gynaecologist. If the NPUI is due to a malignancy, treatment of the malignancy will take precedence.

### 4.7. Future Fertility

There are reports of successful pregnancies following the surgical correction of puerperal uterine inversion. Surprisingly, even though the literature repeatedly says to conserve the uterus if fertility is required, we could not find any evidence of successful pregnancy following repositioning of a nonpuerperal uterine inversion. Irani et al. reported a case where the uterus was repositioned with the Haultain procedure, yet the woman remained subfertile for two years after the operation [143].

Should uterine preservation be successful, there is no evidence to suggest the appropriate interval before attempting pregnancies following these techniques; however, in other recommendations of pregnancy interval following uterine surgery, greater than 12 months is suggested [155]. As with all uterine surgery, the risk of uterine rupture with subsequent delivery should be addressed.

## 5. Conclusions

Nonpuerperal uterine inversion is a rare clinical condition. Due to different presentations of the condition and the low incidence, it can be misdiagnosed on initial assessment. Leiomyomata are the commonest cause of uterine inversion, though a significant proportion was due to malignancies. USS and MRI have been used successfully in confirming the clinical diagnosis. The identification of endometrial tissue after biopsy or laparoscopy would be confirmatory if imaging could not differentiate the condition. Many surgical procedures have been described to treat NPUI, suggesting the diversity of clinical presentation and aetiology. The clinician, depending on the causative pathology, clinical presentation, desire for future fertility of the patient, and the surgical expertise, should select the best surgical approach. Haultain procedure seems to be the most successful method of repositioning the uterus, while the majority of women will require hysterectomy.

## 6. Limitations

The main limitation of the study is related to the rarity of the condition. The absence of clear diagnostic criteria or treatment approaches is evident, and the possible heterogeneity of the approaches reported in this review could be a limitation for the conclusions.

We could find full surgical details among 133 cases out of 153 published cases. Histopathological diagnosis among papers was not consistent, and with the available information, we could categorise only to the subheadings in Table 1.

## Figures and Tables

**Table 1:** Possible etiological factor of nonpuerperal uterine inversions.

	Total number of cases	Leiomyoma	Sarcoma	Carcinoma	Mixed Mullerian tumour	Idiopathic	Others
Thorn in 1911 [4]	96	7 (81%)	1 (1.04%)	4 (4.2%)		13 (13.5%)	
Das in 1940 [1]	54	47 (87.03%)	3 (5.5%)	4 (7.4%)			
Our study^*a*^	153	86 (56.2%)	13 (8.5%)	13 (8.5%)	10 (6.5%)	14 (9.2%)	17 (11.1%)^b^
Total reported cases	303	210 (69.5%)	17 (5.6%)	21 (6.9%)	10 (3.3%)	27 (8.9%)	17 (5.6%)

^a^Cases from 1940 to 2018.^b^13 out of 17 cases were malignancies.

**Table 2:** Stages of uterine inversion.

Stage 1	Inversion of the uterus is intrauterine or incomplete. The fundus remains within the cavity.
Stage 2	A complete inversion of the uterine fundus through the fibromuscular ring of the cervix.
Stage 3	Total inversion, whereby the fundus protrude through the vulva
Stage 4	The vagina is also involved with complete inversion through the vulva along with the inverted uterus

**Table 3:** Surgical options used in the treatment of NPUI.

Surgical detail	Number of cases
*Approach to surgery*	*N* = 133
Abdominal	65 (48.8%)
Vaginal	24 (18%)
Combined abdominal and vaginal	36 (27.1%)
Laparoscopy	8 (6%)

*Succeeded method of repositioning*	*N* = 133
Unsuccessful/not attempted	69 (51.9%)
Haultain procedure	24 (18.0%)
Spinelli procedure	1 (0.8%)
Huntington procedure	1 (0.8%)
Kustner's procedure	8 (6%)
Bisecting the uterus	2 (1.5%)
Repositioned after removing the mass without an additional procedure	9 (6.8%)
Resection of the anterior cervical ring abdominally and repositioned	5 (3.8%)
Others	8 (5.8%)
No details	6 (4.5%)

*The final outcome of surgery*	*N* = 133
Total abdominal hysterectomy/subtotal hysterectomy (with or without abdominal debulking)	53 (39.8%)
Vaginal debulking of the tumour/abdominal hysterectomy	22 (15.8%)
Vaginal hysterectomy (with or without debulking)	26 (19.6%)
Radical hysterectomy and pelvic node dissection	9 (6.8%)
Repair after repositioning (either abdominal or vaginal)	20 (15.0%)
Vaginal amputation of fundus and cervix removed abdominally	1 (0.5%)
Laparotomy/laparoscopic-assisted vaginal hysterectomy	2 (1.5%)
